# Paternal effects on early embryogenesis

**DOI:** 10.1186/1743-1050-5-2

**Published:** 2008-05-16

**Authors:** Laszlo Nanassy, Douglas T Carrell

**Affiliations:** 1 Andrology and IVF Laboratories, University of Utah School of Medicine, Salt Lake City, UT, USA

## Abstract

Historically, less attention has been paid to paternal effects on early embryogenesis than maternal effects. However, it is now apparent that certain male factor infertility phenotypes are associated with increased DNA fragmentation and/or chromosome aneuploidies that may compromise early embryonic development. In addition, there is a growing body of evidence that the fertilizing sperm has more function than just carrying an intact, haploid genome. The paternally inherited centrosome is essential for normal fertilization, and the success of higher order chromatin packaging may impact embryogenesis. Epigenetic modifications of sperm chromatin may contribute to the reprogramming of the genome, and sperm delivered mRNA has also been hythesized to be necessary for embryogenesis. There is less information about the epigenetic factors affecting embryogenesis than genetic factors, but the epigenetics of gamete and early embryogenesis is a rapidly advancing field.

## Background

The contributions of sperm to embryogenesis are more than just providing a haploid genome. In addition to genetic material, sperm factors are involved in many events, such as syngamy, cleavage and epigenetic regulation. There is a growing body of evidence that besides genetic abnormalities, epigenetic abnormalities may be major contributors to idiopathic male infertility and may affect IVF outcome [1]. Male factor infertility is responsible for approximately 50% of in vitro fertilization (IVF) cases and the vast majority of male factor infertility is classified as idiopathic. Therefore, it is important to consider the potential effects of genetic and epigenetic abnormalities on not only male infertility, but also on the outcome of IVF.

**Figure 1:**
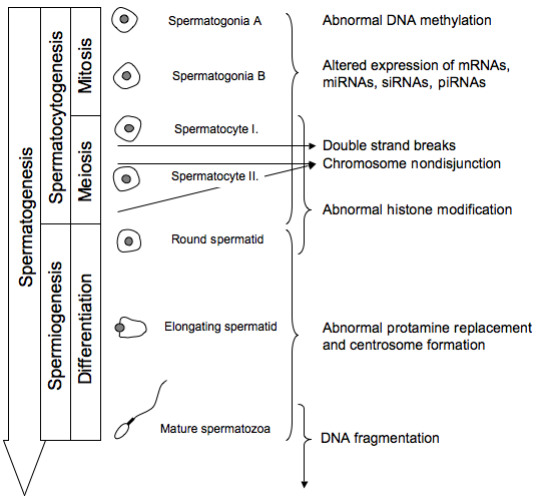
The origin of genetic and epigenetic abnormalities during spermatogenesis. The DNA methylation pattern is established during germ cell development. Spermatocytogenesis can also give rise to chromosome nondisjunction during its meiosis I and II along with double strand breaks, abnormal histone modification and alteration in the expression of mRNA and other non-coding RNAs. Abnormal protamine replacement or centrosome formation can take place at the final stage of spermatogenesis where round spermatids differentiate to mature spermatozoa. DNA fragmentation is mainly the result of apoptosis following double strand breaks or abnormal protamination during spermiogenesis.

ICSI has become a widely used technique to treat many types of male infertility associated with diminished sperm quality. Due to its extensive application there is a growing concern about propagating either genetic or epigenetic disorders. Two such examples are transmission of Y chromosome deletions [2] and a possible elevated incidence of imprinting disorders such as Angelman syndrome or Beckwith-Wiedemann syndrome [3-5]. Here we review the origin of male factors (Figure 1.) that affect the fertilization event and early embryogenesis as well as mechanisms by which these factors have an effect.

### Paternal age

In Western countries, paternal age has increased over the last several decades, with a large number of men fathering children after the age of 50 [6]. Maternal age is an obvious contributor to poor fecundity [7], but little is known about the effect of paternal age. The effect of paternal age on embryogenesis has been measured by investigating the correlation between paternal age and embryo quality, miscarriage rate or pregnancy rate in the general population or in infertile patients. Evaluation of the impact of paternal age on fertility is challenging because older men usually have an older partner, and the exclusion of these couples makes it difficult to reach the proper sample size for evaluation.

An earlier review of the effects of male age on semen quality found that semen volume, sperm morphology, and sperm motility are decreased in men with advanced age but no significant reduction in sperm concentration has been noted [6]. These finding were later confirmed by Ng et al. [8]. Eskenazi et al. studied a recruited healthy population and found a significant age-related decrease in semen quality. The largest age related effect was seen on sperm motility and semen volume, while sperm count was less affected [9]. Age-dependent changes in the testis have been well documented. The number of Leydig cells decrease with age, which leads to a decreased testosterone level and the number of type A spermatogonia is also reduced, and an age-related decrease in testicular tubular lumen has been observed [10].

In IVF cases using donor oocytes from women <35 years of age, the effect of maternal age can be excluded. A recent study showed a significant decrease in the number of day 5 blastocysts in men >55 years old, but no difference was observed in earlier developmental stages. A higher pregnancy loss and decreased live birth rate has also been reported in men over the age of 50 [11]. Watanabe et al. showed a 10% decrease in fertilization rate with semen from men ≥ 39 years old compared to those who were under 39. When the number of donated oocytes was 5 or less, the clinical pregnancy rate was also reduced in couples with an older male partner [12]. However Gallardo et al. and Paulson et al. saw no effect on pregnancy rates [13,14].

The effect of paternal age on pregnancy outcome following natural conception has been investigated in population-based studies. In a prospective study, a significantly higher spontaneous abortion rate was found in women with male partners older than 45 years compared to those whose partners were less than 25 years of age [15]. Kleinhaus et al. reported that partners of women 40 years or older had an almost three-fold increase in spontaneous abortion compared to those women with a partners 25 years old or younger [16].

Analyzing IVF cycles, a negative effect of advancing paternal age on pregnancy and live birth rate after IVF and gamete intrafallopian transfer has been reported [17]. In another study in which IVF data from 1,938 couples were retrospectively analyzed, an increased risk of conception failure was noted in men over 40 years old [18]. However, several reports indicate that paternal age does not affect pregnancy outcome in ICSI cycles [19-21].

The possible reason behind these results could be increasing sperm DNA fragmentation with age [22], possibly due to a decrease in apoptotic activity during spermatogenesis [23]. It has been hypothesized that sperm aneuploidy is also a contributor to pregnancy loss among partners of aging men [24], however an exact relationship between aging and sperm aneuploidy has not been established. A significantly higher frequency of chromosome aberrations was found in men older than 59 compared with men younger than 40 [25], but it has not been confirmed by another group. They found no age-related change in the incidence of sperm aneuploidy [22].

The current body of data suggests advancing paternal age is associated with decreasing sperm characteristics that may reduce the chance for fathering, particularly in men over 50 years of age [6,9]. While still not definitively demonstrated, IVF outcome may be moderately affected by paternal age [26].

### The source of sperm

The effect of sperm source on embryogenesis has been investigated in a limited number of retrospective studies. An early study by Nagy et al. concluded that using fresh and frozen-thawed epididymal and testicular spermatozoa from obstructive azoospermic patients gave results that were comparable to those obtained from ejaculated spermatozoa following ICSI [27]. Similar results using ejaculated sperm compare to epididymal or testicular sperm from patients with obstructive azoospermia has also been reported, however lower fertilization and pregnancy rates were observed using testicular sperm patients from non-obstuctive azoospermia [28]. Another group also reported higher fertilization rate using ejaculated sperm compared to testicular sperm from non-obstructive azoospermic men, but further differences were not observed in the developmental data [29].

Some data indicate that patients with obstructive azoospermia can achieve higher fertilization, implantation and birth rate compare to those who have non-obstructive azoospermia [21,30]. Similarly, higher fertilization and implantation rates have been observed comparing obstructive to non-obstructive azoospermic patients, but no difference was found in the pregnancy rate [31]. Pasqualotto et al. find similar fertilization rate between obstructive and non-obstructive azoospermic patients but significantly reduced pregnancy and elevated abortion rates [32]. There are indications that non-obstructive azoospermic men have higher frequency of aneuploid sperm [33-35], which can lead to miscarriage [36].

Studies investigating the effect of sperm recovery sites in obstructive azoospermic men found contradictory results. No difference was observed in fertilization, pregnancy rates [28] or birth rate when epididymal or testicular sperm were used [21]. Another study reported a higher implantation rate using testicular sperm over epididymal sperm, however the pregnancy rate was not significantly higher [37]. Buffat et al. also found a significantly higher miscarriage rate using testicular spermatozoa than epididymal spermatozoa and concluded that this is probably due to the affect of immaturity of testicular spermatozoa on embryogenesis [38]. Regardless of the etiology of azoospermia, similar pregnancy rate was reported using testicular or epididymal sperm, but lower miscarriage rate has been observed following ICSI using sperm retrieved from the epididymis [39]. These studies suggest that the fertilization and pregnancy rate is not compromised using testicular biopsy-derived sperm from obstructive azoospermic patients compared to using ejaculated sperm.

### Genetic factors

#### DNA fragmentation

DNA fragmentation has been associated with altered reproductive outcome, although the rate of the correlation varies between studies using different methods to detect DNA damage [40]. DNA fragmentation is characterized by single and double strand breaks, which are the result of three main sources: abortive apoptosis, oxidative stress, and general abnormalities in the process of recombination and protamination [40,41].

The key indicator of apoptosis is DNA strand breaks, which, however, could also be unrelated to apoptosis, since they are the result of normal processes as well, such as chromatin remodeling or protamine replacement during spermatogenesis [42]. Oxidative stress is the result of the production of reactive oxidative species (ROS) and insufficient antioxidant activity. Antioxidants along with the highly compact structure of sperm chromatin are the only defense mechanisms against free radicals [40,43]. Spermatozoa are highly vulnerable to oxidative stress, since they are transcriptionally inactive and have only a small amount of cytoplasm, which lacks both antioxidants and a DNA-repair system [44]. Improper compaction due to an altered protamine ratio has been shown to result in significantly higher DNA damage, which confirms a strong correlation between protamination and DNA integrity [45].

Several methods have been used to detect DNA fragmentation such as sperm chromatin structure assay (SCSA), terminal deoxynucleotidyl transferase-mediated dUDP nick-end labeling (TUNEL) and single cell electrophoresis (Comet) assay [40,42,46,47].

High DNA fragmentation is associated with diminished sperm count, motility, and morphology [22,48,49]. Decreased fertilization and implantation rates associated with increased DNA fragmentation have been reported [49]. A negative correlation was observed between the extent of DNA damage and developmental rate to the blastocyst stage after IVF or ICSI [47,50]. An increased incidence of sperm DNA fragmentation has also been associated with higher spontaneous abortion rate [51] or unexplained recurrent pregnancy loss [52].

Different DNA damage test provided different results in relation to embryo development and pregnancy loss between ICSI and IVF cases. DNA fragmentation detected by TUNEL assay has been associated pregnancy loss in ICSI cases while this relationship has not been established in IVF cases [53]. Also in ICSI cases embryo cleavage was negatively correlated to DNA fragmentation measured by Comet assay [54]. SCSA test might be less sensitive detecting differences between IVF and ICSI cases [55-57]. To define the exact relationship between these tests, their results and IVF outcome more investigation is needed. It seems the predictive value of DNA fragmentation for unsuccessful IVF treatment cycle is stronger in ICSI cases than in IVF cases.

Elevated DNA fragmentation in sperm from infertile patients is a common pathology. Detection of DNA damage can be advantageous especially for patients with idiopathic infertility, however the exact phenotype that can benefit from it is still under investigation.

#### Sperm aneuploidy

Regardless of the chromosomes involved, most embryonic aneuploidies are of maternal origin, most commonly as a result of a meiosis I error. However, a significant paternal contribution to sex chromosome trisomies has been shown [58,59].

Sperm morphology is normally not related to aneuploidy, except in men especially with a high percentage of macrocephalic, multinucleated, and multiflagellate sperm or severe oligoasthenoteratozoospermia show an increased incidence of sperm aneuploidy [60-64]. Severe oligozoospermia is also associated with higher aneuploidy rate than normal fertile donors [35]. Nagvenkar et al. also showed that patients with severe oligozoospermia have higher frequency of XY and YY disomy than oligozoospermic and normospermic men [65].

The importance of sperm aneuploidy is obvious given the fact that spermatozoa with numerical chromosome abnormalities are able to fertilize an oocyte, resulting aneuploid concepti [66]. In a case report it has been reported that in case of two live-birth (47, XXY and 47, XYY) and two spontaneously aborted (47, XX + 15 and 47, XX + 22) pregnancies the father had elevated frequency of disomic sperm for every chromosome involved in trisomic pregnancies [67]. An increased sperm aneuploidy rate has been associated with lower implantation and pregnancy rates and a higher incidence of miscarriage in ICSI cycles [36,65,68,69].

The most commonly used technique for evaluating aneuploidy in sperm samples is fluorescent in situ hybridization (FISH), however there are techniques on the horizon, such as array based comparative genomic hybridization (aCGH), yielding a more complex assessment [70]. The evaluation of homologous chromosome recombination at prophase I of meiosis has recently gained attention. Meiotic crossover is important for two reasons; first it contributes to genetic variability; and second it provides a physical connection for the proper segregation of chromosomes. Each bivalent must contain at least one recombination site. In the absence of a crossover, nondisjunction can occur. Smaller chromosomes, which usually have only one crossover such as chromosomes 21 or 22, have a higher frequency of aneuploidy [71-73].

The number and position of recombination foci on individual chromosomes in spermatocytes can be determined by the immunostaining of elements in the synaptonemal complex. Specifically, staining of MLH1, a marker of crossover events, and CREST, which attached to the centromere, allows for both chromosomal enumeration and the localization within the synaptonemal complexes. Studies have demonstrated that infertile men either with nonobstuctive azoospermia or obstructive azoospermia show a lower frequency of recombination and a higher number of cells with at least one bivalent without crossover than fertile controls. An increased number of gaps and splits in the synaptonemal complexes are also observed in infertile patients [74,75]. Lower rates of recombination between chromosomes are associated with a higher risk of producing disomic sperm [76].

These studies suggest that more care has to be taken with those patients who have severe oligoastenoteratozoospermia, non-obstructive azoospermia or unexplained recurrent pregnancy lost. The aneuploidy screening is highly recommended for patients with these etiologies. Aneuploidy screening an expensive and a very time consuming process required about 10 hours of technician time. Using automated analysis system might provide a solution since it could reduce time by one third although it is still costly [77]. If elevated frequency of aneuploidy confirmed, these patients must be counseled for the elevated risk of miscarriage or conceiving aneuploid offspring.

### Epigenetic factors

#### Centrosome functions

In most mammals, including humans the centrosome is inherited from the fertilizing sperm. During spermatogenesis, centrosome reduction takes place resulting a single centriole and reduced amount of pericentriolar material in spermatids. In order to avoid abnormal fertilization the maternal centrosome is fully degradeted, however proteins that are essential for normal centrosome function, such as γ-tubulin, during embryogenesis are maintained in the oocyte [78-80]. After fusion of the gametes, the aster forms from the sperm centrosome synchronously with sperm head decondensation and the arising microtubule organizing center (MTOC) promotes the proper positioning of the male and female pronuclei. The paternal centrosome then duplicates during the first cell cycle to ensure cleavage later on [78,81,82].

The transfer of a normally formed centriole to the oocyte by the spermatozoa is essential for proper fertilization. Malformation of the centrosome is associated with detrimental phenomenon at fertilization such as abnormal sperm aster formation which leads to the failure or lack of syngamy resulting in cleavage arrest [83]. Improper centrosome function may also lead to numerical chromosomal abnormalities causing aneuploidy or mosaicism in the embryo [84,85].

Centrosomes were usually assessed by transmission electron microscopy, however the technology is difficult, laborious to carry out and provides information only about the morphology of the centrosome [86] whereas normal morphology does not always indicate proper functionality. Microinjection of bovine, rabbit oocytes with human sperm allow the immunohistochemical study of aster formation [87-89]. This functional assay is proven be a useful tool in studying abnormal centrosome function in some cases, [83,89] although the wide-range clinical application is unlikely due to the complexity of the method.

#### Chromatin packaging

In human sperm, two protamines are expressed protamine 1 (P1) and protamine 2 (P2). Protamines replace nuclear histones (H2A, H2B, H3 and H4) in a stepwise manner, resulting in highly condensed and transcriptionally silent chromatin during sperm maturation. In round spermatids the chromatin structure is similar to that observed in somatic cells. During spermiogenesis nuclear histones become hyperacetylated and shortly there after disassembly and replacement by testis-specific histone variants followed by transition proteins (TP1 and TP2) occurs. At the final stage of spermiogenesis, removal of transition proteins takes place followed by protamine replacement. In a mature human spermatozoon, 85% of histones are replaced by protamines [43,90,91].

In humans, P1 and P2 are expressed in nearly equal quantities, with the P1/P2 ratio close to 1, alteration of the protamine ratio in either direction is associated with diminished sperm parameters. While altered protanime ratio has never been observed in fertile men [91,92]. Abnormal P1/P2 ratio is associated with low sperm count, reduced motility, abnormal head morphology, higher frequency of DNA fragmentation and lower sperm penetration assay score [48,93]. Reduced fertilization rate has also been observed in these patients following IVF cycles, but ICSI results in normal rates. Altered P1/P2 ratio is likely incompatible with the early events of the fertilization process such as capacitation, acrosome reaction, membrane fusion or penetration, but this can be overcome using ICSI. Protamine deficiency evaluated by chronomycin A3 (CMA3) may also correlate to fertilization failure following ICSI because of premature sperm chromosomal condensation (PCC) [94]. Decreased clinical-pregnancy rates have been shown following fertilization with sperm having reduced P1/P2 ratio [95]. Aberrant P1/P2 ratios can be the result of either P1 or P2 deregulation, however the majority of cases show P2 deregulation [93]. Normal sperm function requires a high order of chromatin packaging. The protective function of the tight protamine packaging against endogenous and exogenous agents such as nucleases, free radicals or mutagens has also been emphasized by different authors [48,91]. Further studies are needed to ascertain any potential impact of abnormal chromatin packaging on the development of the resulting early embryo.

#### Epigenetic modifications

##### Histone modification

Histones are the best candidates for the transmission of epigenetic information because of their influence on the modification of chromatin structure, which modulates access of the machinery to the genes in an organized pattern [96,97]. Gene activity is determined by methylation, acetylation, ubiquitylation and phosphorylation of histones depending on the nature and position of modification of the amino acid involved [98]. Histone acetylation is associated with transcriptional activity, which is regulated by the activity, concentration, interaction and availability of cofactors of histone acetyl transferases (HATs) and histone deacetylases (HDACs).

Histone methylation is a complex process that confers a high degree of specificity in the regulation of gene expression. In spermatogenesis, histone methylation is carried out by the H3-K4 and H3-K9 methyltransferase families. These methylatransferases facilitate gene silencing by mono-, di- or trimethylation of lysine or arginine [99,100]. The number and location of methyl groups on the sperm-specific histones is one component of gene regulation in spermatogenesis. It has yet to be determined if these modified histones play a crucial role in gene expression during early embryogenesis or whether abnormal histone modifications in the sperm are associated with diminished embryo development as well.

##### DNA methylation

DNA methylation is another major epigenetic factor that regulates the function of the genome along with histone modification. The methylation of DNA is catalyzed by DNA methyltransferases, namely DNMT1, which restore the DNA methylation pattern of CpG islands following DNA replication. DNMT3a and DNMT3b are responsible for establishing *de novo *DNA methylation. DNA methyltransferases transfer a methyl group to CpG dinucleotide residues in order to regulate gene activity. Hypomethylation is associated with transcriptional activity while hypermethylation is associated with gene silencing through methyl-CpG-binding proteins, which contain a transcription repression domain [99,100].

During germ cell development the whole genome is demethylated, which erases parental imprinting marks and the newly establish methylation pattern results in the resetting of imprints. The maternal and paternal DNA are methylated in a parent-of-origin-dependent manner, resulting in a different expression pattern of imprinted genes between genders. Following fertilization, genome-wide active demethylation of the paternal genome takes place, then both the paternal and maternal genomes undergo demethylation by a passive mechanism with the exception of some regions such as imprinted genes, restoring the totipotency to the fertilized egg. After implantation remethylation of the embryonic genome takes place, establishing a hypermethylated ICM compare to the trophoblast [100-104].

Alterations in methylation patterns can result in biallelic expression or repression of imprinted genes and can cause malformation [105]. More than half of the cases of Beckwith-Wiedeman syndrome and around 10% of the cases of Angelman syndrome are associated with epigenetic defects. These syndromes appear to occur at a higher incidence following ICSI [3-5]. A method for measuring the global methylation status of sperm DNA by immunostaining of 5 methyl-cytosine was reported by Benchaib et al. In their preliminary study, the DNA methylation level was decreased in the sperm of patients with asthenozoospermia and teratozoospermia compared to that from normal ejaculate [106]. In another study a correlation was found between global mehylation level and pregnancy rates [107]. No differences in the global DNA methylation patterns were seen in protamine-deficient sperm samples indicating that this assay might not sensitive enough to detect variation in epigenetic modification [108]. More informative techniques such as bisulfite genomic sequencing or CpG island microarrays [99] should be used to analyze methylation patterns of imprinted genes rather than global DNA methylation to identify possible differences in infertile males. Categorization of infertile men by a more detailed analysis of DNA methylation pattern might reveal a new origin of reduced fertilization, implantation or pregnancy rates. However the application of this technique in a clinical setting in the near future cannot be expected.

##### RNA-associated silencing

Small noncoding RNAs (ncRNA) have recently been associated with epigenetic modifications [109]. A suite of noncoding RNAs, probably micro RNAs (miRNAs) and small interfering RNAs (siRNAs) has been identified in human sperm [110]. Recently another type of interfering RNA called PIWI interacting RNAs (piRNAs) has been described in mouse. These are longer than miRNAs or siRNAs and found to bind to PIWI [111]. Whether these noncoding RNAs have an effect on embryogenesis and if so, what their role is, are intriguing questions to answer in the future.

#### mRNAs

It has long been thought that spermatozoa are transcriptionally quiescent. Despite this, it was found that mature spermatozoa contain fully processed mRNA, albeit in a much lower amount compared to mature oocytes [111-114]. First it was assumed that these mRNAs were leftover from spermatogenesis, but there is evidence that the spermatozoa delivers a unique set of mRNAs to the oocyte at fertilization that have been hypothesized to be necessary for proper embryogenesis. Furthermore, the fact that some of these spermatozoal mRNAs are also found in zygotes indicates these transcripts may be functionally important [115,116]. The spermatozoal mRNA fingerprint of individuals can be identified by microarray analysis. Any alteration in the amount or composition of sperm mRNAs may indicate abnormalities in spermatogenesis [116]. It has been already shown that mRNA fingerprint differs between normospermic and teratozoospermic men [117]. This finding supports the idea that perturbation in the transcription in late spermiogenesis may affect embryogenesis. The correlation between the sperm mRNA fingerprint and embryogenesis needs further clarification in the future.

## Conclusion

Since ICSI became a treatment option for many type of severe male factor infertility including azoospermia, there is a growing concern about the safety of the treatment. The fertilizing spermatozoon is associated with more functions than only providing half of the genetic material to the oocyte. There are several factors that should be considered choosing the proper treatment option.

The integrity of the haploid set of chromosomes is highly important. The recognition of genetic abnormalities such as DNA fragmentation or sperm aneuploidy is already possible, however the routine clinical application remains limited. The characterization of patients who can benefit from it is obvious in some cases however needs further investigation. Using sperm for IVF treatment from these patients can lead to reduced fertilization, diminished implantation and pregnancy rates or production of aneuploid offspring. Since ICSI has been extensively used to treat many kinds of male infertility, there is higher risk for propagating these heritable anomalies to the future generations.

Recently, the investigation of the contribution of the spermatozoa to early embryogenesis beyond the genetic factors has gained attention. There is evidence of epigenetic contribution to complex diseases. Epigenetic factors mentioned above likely contribute to embryogenesis since it is believed that the totipotency of the embryo is the result of genome wide demethylation. DNA methylation and histone modification are acting together. Abnormalities in either or both functions might result to diminished fertilization, disturbed embryogenesis or reduced implantation and pregnancy rates. Studies evaluating epigenetic factors in relation to infertility or embryogenesis are in its infants. More effort should be taken in order to study the clinical aspects of epigenetic abnormalities.

More information is sorely needed to evaluate the risk of transmission of genetic or epigenetic defects in order to offer safer infertility treatments. The first step is the wider application the existing knowledge by developing reliable, cheap and easy to use assays for clinics. Extensive research is ahead to developing more informative assays in relation to identify paternal factors, as well as the mechanisms that they are involved in early embryogenesis.
